# Oxidation states of copper in preservative treated wood as studied by X-ray absorption near edge spectroscopy (XANES)

**DOI:** 10.1371/journal.pone.0263073

**Published:** 2022-01-27

**Authors:** Samuel L. Zelinka, Grant T. Kirker, George E. Sterbinsky, Keith J. Bourne

**Affiliations:** 1 Building and Fire Sciences, US Forest Service, Forest Products Laboratory, Madison, WI, United States of America; 2 Durability and Wood Protection, US Forest Service, Forest Products Laboratory, Madison, WI, United States of America; 3 Advanced Photon Source, Argonne National Laboratory, Lemont, IL, United States of America; Argonne National Laboratory, UNITED STATES

## Abstract

Copper is a common component in wood preservatives and is used to protect the wood against fungal degradation. Previous research has shown that the Cu^++^ oxidation state provides the best wood protection, and Cu^++^ is widely believed to be the oxidation state of most copper within treated wood. A recent study using X-ray absorption near edge spectroscopy (XANES) reported high amounts of Cu^+^ in wood that had been in contact with corroded fasteners. This study uses XANES to examine the copper oxidation states in wood treated with several different wood preservatives as a function of time after treatment. In contrast with previous literature which focused on the fixation reaction in the first few hours after treatment, this paper examines the oxidation state of Cu in treated wood at longer times (up to 1-year) after treatment. The results showed in nearly all cases, Cu was in the Cu^++^ oxidation state to within the measurement uncertainty. Cu XANES patterns taken approximately 1-year after treatment showed no discernable differences between preservative systems, indicating that regardless of the starting treatment the final Cu speciation is the same within one year. The results confirm previously held beliefs about the Cu oxidation states in wood and give further insights into the corrosion mechanism of metals embedded in treated wood.

## Introduction

Wood is susceptible to biodegradation when it is placed in outdoor or high moisture environments. Wood preservatives are chemicals that are used to extend the service life of wood in these challenging environments. In the United States, most wood preservatives contain copper as one of the primary chemicals to protect the wood against fungi. Copper has been used in wood preservatives since the early 1800s [1] and is still a major component of nearly all standardized wood preservatives used to treat wood in direct contact with the ground [2].

For many years, Chromated Copper Arsenate (CCA) was the most widely used wood preservative throughout the United States and constituted nearly the entire market for treated lumber. However, in 2004, CCA was voluntarily withdrawn for use in the US. Following the withdrawal of CCA, many new wood preservatives were introduced to the market [3]. Two of the most commonly used preservatives were Alkaline Copper Quaternary (ACQ) and Copper Azole (CA). These preservatives both contained more copper than CCA, and it was observed that these preservatives increased the corrosion of embedded fasteners, sometimes causing structural collapse [4–7].

In response to corrosion concerns observed with ACQ, and CA, the wood preservative industry developed “micronized” copper preservatives [8]. For traditional, soluble copper systems such as CCA, ACQ, and CA, the copper is dissolved into the treatment solution. Micronized systems pulverize copper carbonate into sub-micron nanoparticles which are suspended in the treatment solution. Most of the nanoparticles are between 10 nm and 700 nm in size, with the median particle size reported to be between 100–200 nm in length [9]. Micronized copper systems are standardized in the American Wood Protection Association (AWPA) standards [10], and also governed by International Code Council Evaluation Reports. Micronized systems commonly have a traditional soluble copper counterpart with the same co-biocide. For instance, Micronized Copper Azole (MCA) is the micronized copper system analog of CA, and Micronized Copper Quaternary (MCQ) is the micronized counterpart of ACQ. These micronized systems now account for at least 80% of the pressure treated wood sold for residential construction [9].

There has been much research on wood treated with micronized systems over the past 10 years. Since these preservatives are introduced to the wood as particles rather than in a soluble solution there is concern that they do not penetrate the wood cell wall as well, especially in refractory species [11, 12]. In a recent review, Tarmian [13] concluded that “micronized copper-based preservatives cannot penetrate as deeply or uniformly as the soluble copper-based ones”. The distribution of copper in the cell wall with micronized systems has been studied and it has been found that copper particles aggregate in bordered pits, rays, and resin canals [14, 15].

In addition to studies on micronized wood treatments, several researchers have examined whether wood treated with these preservatives emit nanoparticles into the environment as these wood treatments represent one of the largest industrial uses of nanoparticles in any industry [16]. Parks et al. [17] found that micronized systems leached less copper into water than soluble systems but did not examine particle size. Lankone et al. [18] observed that the much of the released copper was bound to wood particles; however, 45% of the copper emitted was in nanoparticle form between 20–240 nm. Finally, work by Johnson et al. [9] used micro-X-ray absorption near edge spectroscopy (µXANES) to study copper emitted by treated wood and observed that much of the copper cycled to a soluble Cu^++^ state in the soil and even in the wood at long enough times.

Regardless of whether the preservative is a traditional or micronized formulation, most of the copper in the treated wood is believed to be in the Cu^++^ state. Divalent copper ions are more toxic to fungi than cuprous ions or copper metal [19], however cupric ions in wood can also lead to the corrosion of metal fasteners [20]. Understanding the copper oxidation states and copper binding with the wood cell wall is incredibly important for developing effective wood preservatives that are not corrosive. Most of what is known about the oxidation states of treated wood comes from electron paramagnetic resonance (EPR) measurements on freshly treated wood. These experiments focused on the first hours after treatment and observed how the chemicals reacted with and bonded to the wood, namely how the Cu ligands changed [21–25]. However, since this technique can only measure paramagnetic ions, it is only able to observe the binding of cupric ions and cannot directly assess whether Cu^+^ plays an important role in the preservative system. In corollary experiments, Xue et al. [25] compared the amount of Cu^++^ measured with EPR to the total amount of copper measured with X-ray fluorescence spectroscopy and determined that 95% or more of the copper in treated wood existed in the Cu^++^ oxidation state.

**Fig 1:**
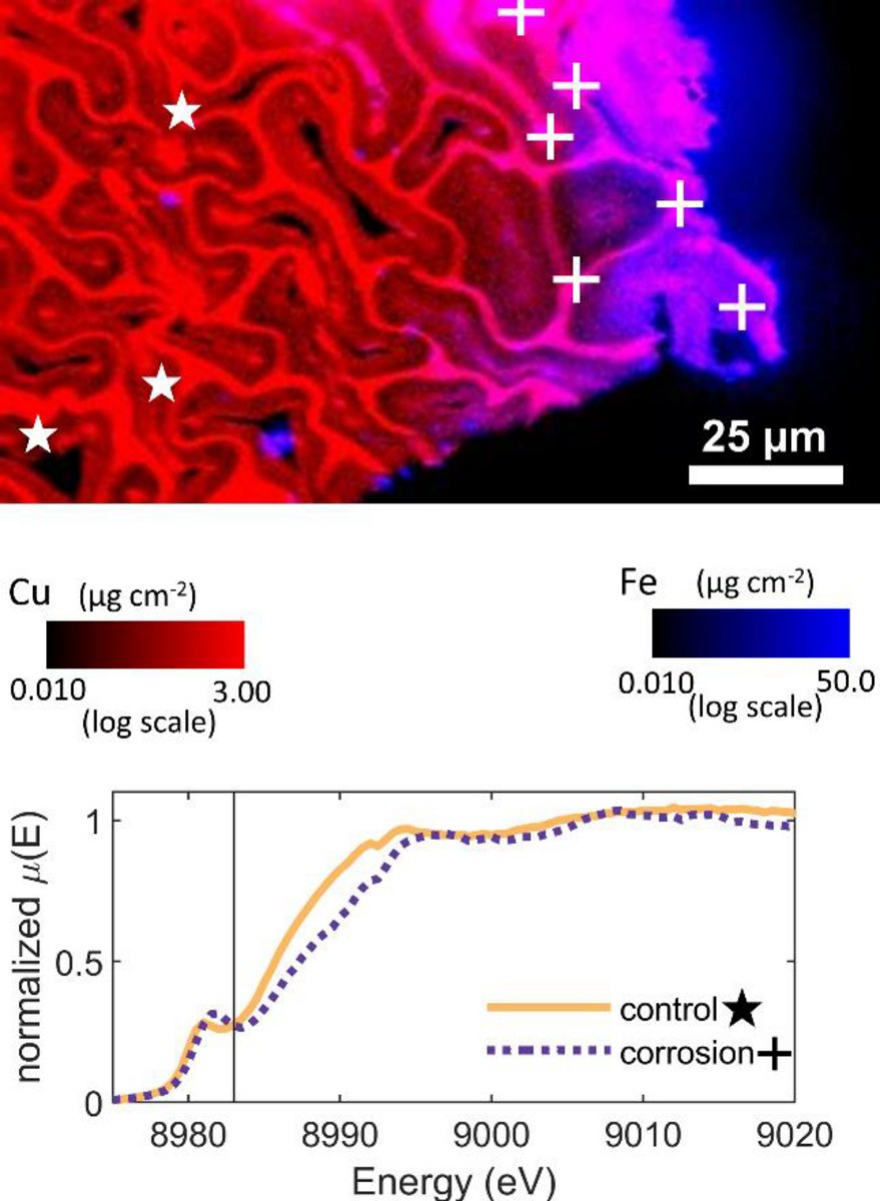
XFM map of the copper and iron concentrations showing the µXANES scan locations in the "control" (★) and "corrosion" (+) regions for MCQ treated wood in contact with a steel fastener for 1 year; the normalized X-ray absorption coefficient is provided for each group at the bottom of the figure. (Note, the lines presented represent the mean *μ*(*E*) at each energy for all locations sampled). The signal at 8983 eV (vertical line) is proportional to the amount of Cu+ in the sample, see “Calculation of Copper Oxidation States”.

Recent measurements have cast doubt upon the amount of Cu^++^ in treated wood. Zelinka et al. [26] examined treated wood adjacent to corroded fasteners to better understand the corrosion mechanism in treated wood. They used both X-ray Fluorescence Microscopy (XFM) to map out the copper concentrations within the first 200 µm of the fastener surface and µXANES to measure the oxidization states in these areas. Example data for wood treated with MCQ is given in Fig 1. In Fig 1, the copper concentration is plotted on a color scale using red, and that of iron is shown with blue; the cells on the far right were exposed to the fastener surface. The XFM map shows a purple region near the fastener surface caused by iron corrosion products diffusing into the wood. Zelinka et al. [26] designated this region the corrosion affected zone and compared these measurements against those taken farther (hundreds of micrometers) from the fastener surface (labeled control in Zelinka et al. [26] and Fig 1). However, both measurements showed that more than 2/3rds of copper was in the Cu^+^ oxidation state [27] (bottom of Fig 1).

The measurements of Zelinka et al. [26] are in contrast to the majority of the literature on wood preservation. Not only are they the only XANES measurements on copper in treated wood, they are also the only measurements of the copper oxidation states taken a long time after the preservative treatments. Therefore, the differences between these measurements and what is known about copper in treated wood from fixation experiments require further examination.

Differences in the amount of Cu^++^ in the wood may arise from differences in the conditions of the experiments, such as the long exposure duration and proximity to corroding fasteners in the work of Zelinka et al. [26]. It is also possible that differences in experimental techniques led to the discrepancy in the literature. The XANES measurements of Zelinka et al. [26] may have been affected by beam damage, which has been shown to reduce Cu^++^ to Cu^+^ [28] although Zelinka et al. did not examine that in their study.

In this study, we investigate copper oxidation states in wood treated 1 day to over 1 year prior to measurements, including samples that had been included in field exposures. The goal is to understand whether copper in treated wood undergoes reduction at long times and better understand the results of Zelinka et al. [26] who observed high amounts of Cu^+^ in treated wood.

## Methods

Four different preservatives were examined: Chromated Copper Arsenate type C (CCA), Copper Azole (CA), Micronized Copper Azole (MCA), and Alkaline Copper Quaternary (ACQ). These formulations were chosen so that they could be compared against the measurements of Zelinka et al. [26, 27], which found high amounts of cuprous ions (68–83% of total copper) in treated wood from a corrosion test. The formulations of CCA, CA, ACQ and MCA are specified in AWPA standards P23, P48, P29 and P61, respectively, [29–32] and the formulation of MCA is also given in an ICC-ES Evaluation Report (ESR 2240) [33]. The composition and loadings of the preservatives are given in Table 1.

**Table 1:** Preservatives examined in this study along with their composition and total retention.

Abbreviation	Preservative Name	Composition	Retention (kg m^-3^)^a^
CA	Copper Azole	96.1% CuO	1.0
		1.95% Propiconazole	
		1.95% Tebuconazole	
MCA	Micronized Copper Azole	96.1% CuO	1.0
		3.9% Tebuconazole	
CCA	Chromated Copper arsenate	47.5 wt.% CrO_3_	4.0
		18.5 wt.% CuO	
		34.0 wt.% As2O_5_	
Samples from Zelinka et al. [26] re-analyzed in this study			
ACQ	Alkaline copper quaternary (Type D)	66.7 wt.% CuO	2.9
		33.3 wt.% DDAC^b^	
MCQ	Micronized copper quaternary	66.7 wt.% CuO	5
		33.3 wt.% DDAC	
CA	Copper Azole (Type C)	96.8 wt.% CuO	0.7
		1.6 wt.% Tebuconazole	
		1.6 wt.% Propiconazole	
CCA	Chromated copper arsenate (type C)	47.5 wt.% CrO_3_	1.6
		18.5 wt.% CuO	
		34.0 wt.% As2O_5_	

^a^Determined by ICP [10], except CA and MCA which were indicated by end tag retentions.

^b^DDAC, didecyldimethylammonium carbonate.

Southern pine (*Pinus spp*.) blocks (33m x 26mm x 5mm) were treated by submerging the blocks in a treatment solution and subjecting to vacuum (27mm/Hg) for 30 min. The blocks were kept submerged in the treatment solution and then removed at intervals and allowed to dry under laboratory conditions. Samples were removed at various time points so the time for fixation ranged from 1 day to 1 month before X-ray measurements. Additional samples were examined that were removed from an above-ground field test after 1 year. Finally, the parent blocks used to prepare the 2 µm thick samples of Zelinka et al. [26] were reanalyzed in this study at the macroscale.

XANES measurements were taken at beamline 9-BM-C at the Advanced Photon Source (APS) at Argonne National Laboratory. The approximate diameter of the beam was 1 mm. Wood samples were affixed to a sample holder without any other sample preparation, and the XANES patterns were collected using a 4-element Vortex ME4 fluorescence detector. Count rates were less than 82,000 per detector element per second. The beam penetrated depth was calculated to be between 1.5 and 4.5 mm (depending upon the wood moisture content) using mass absorption coefficients taken from Hephaestus [34]. As a result, the XANES patterns are most strongly sampling the copper within the first few millimeters of the wood surface.

Measurements were taken from 200 eV below the copper *K*-edge of 8980.48 eV to 547.53 eV above. The size of the energy step depended upon the distance from the *K*-edge. Data points were acquired in 5 eV steps from -200 to -20 eV, 0.3 eV steps from -20eV to 45 eV, and steps with progressively increasing size for energies greater than 45 eV above the *K*-edge. At each energy, an integration time of 0.5s was taken before moving to the next energy. The X-ray absorption coefficient (*μ*(*E*)) was calculated by normalizing the fluorescence intensity by the upstream ion-chamber current in the Athena software package (version 0.9.22) [34]. Pre-edge and post-edge background removal were then performed using the Athena software to yield a normalized *μ*(*E*).

### Calculation of copper oxidation state

**Fig 2:**
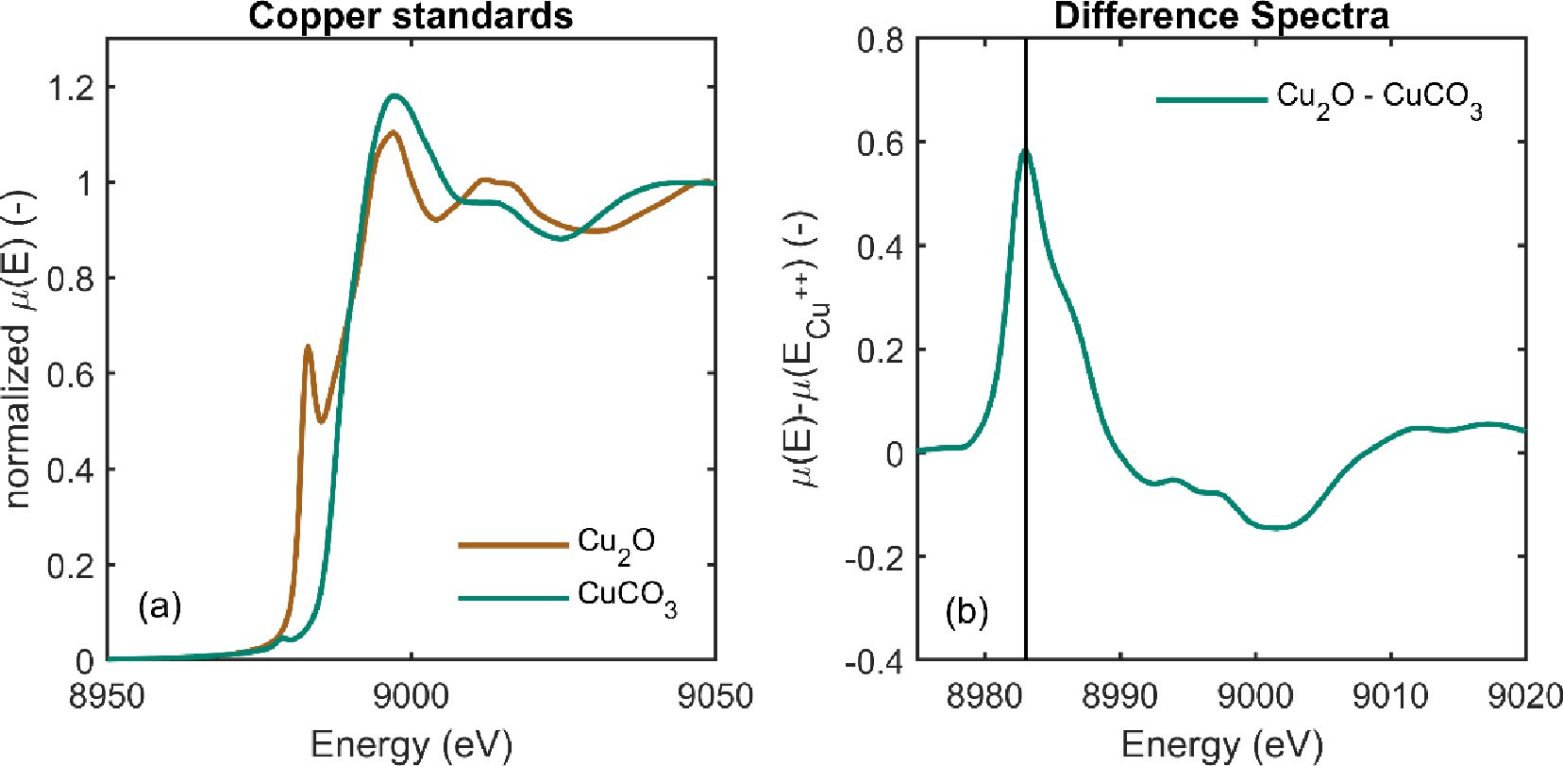
(a) Cu+ and Cu++ standards used to evaluate oxidation states in the study. (b) Difference spectra. The vertical line is at 8983eV and is the energy used to determine the fraction of Cu^+^ in the unknown samples.

Copper oxidization states were determined using the method of Kau et al. [35]. This method takes advantage of the fact that the Cu^+^ XANES pattern has a peak at 8983 eV that does not appear in the Cu^++^ spectrum (see Fig 2A). Therefore, to determine the amount of Cu^+^ in a sample (*f*_*c*1_), the intensity of an unknown sample is compared against the difference between the Cu^+^ and Cu^++^ standards at 8983 eV, i.e.

$$f_{C1}=\frac{\mu (E_{x})-\mu (E_{2})}{\mu (E_{1})-\mu (E_{2})}$$ (Eq 1)

where *μ*(*E*_1_) is the normalized X-ray absorption coefficient of the Cu^+^ standard at 8983 eV, *μ*(*E*_2_) is the normalized X-ray absorption coefficient of the Cu^++^ standard at 8983 eV, and *μ*(*E*_*x*_) is the normalized X-ray absorption coefficient of the unknown sample at 8983 eV. Note that for some treated wood spectra Eq 1 predicts an unphysical negative value of Cu^+^, whose maximum magnitude was 4%. Therefore, the uncertainty in the technique is at least 4%, but could be larger.

## Results and discussion

### Behavior over the first year after treatment

**Fig 3:**
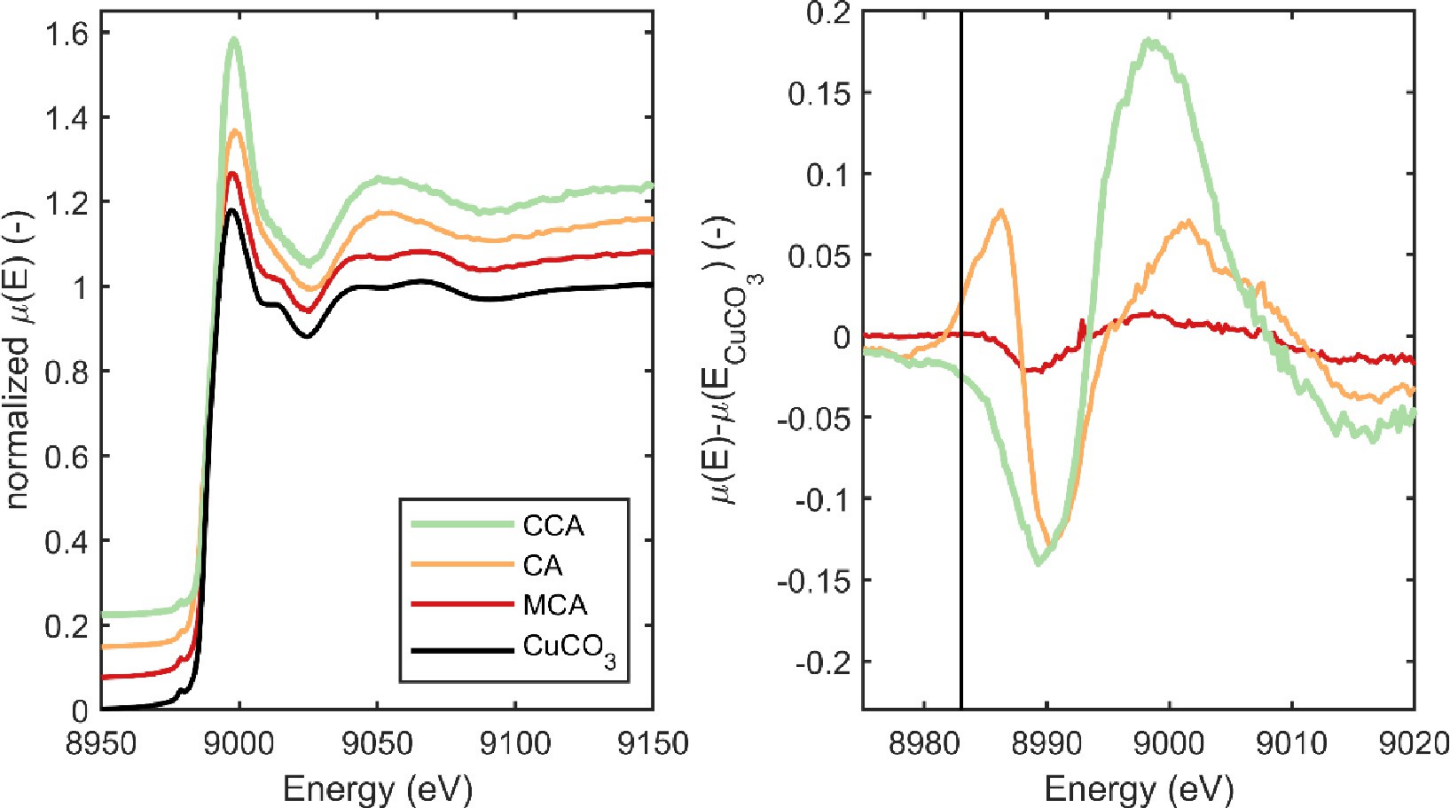
(a) XANES spectra of the preservative systems examined 1-day after treatment compared to the CuCO_3_ standard. (b) Difference spectra between the normalized *μ*(*E*) data and the CuCO_3_ standard; the vertical line is at 8983 eV and used to calculate E_x_ in Eq 1.

Fig 3 shows the XANES patterns of the preservatives 1 day after being removed from the treating solution along with the CuCO_3_ standard. Fig 3A clearly shows that there is little evidence of a Cu^+^ peak at 8983 eV in any of the experimental treatments. Furthermore, the general shape of the XANES patterns resemble that of the CuO and CuCO_3_ standards. While these spectra are useful for observing general trends in the data, more information can be seen in the difference spectra (Fig 3B). In Fig 3B, the three preservatives exhibit very distinct behavior. The MCA shows very little deviation from the CuCO_3_ standard (Fig 3B). CCA and CA residuals plotted in Fig 3B. exhibit several deviations from the baseline and are distinct from each other. Therefore, while all preservatives are comprised almost entirely of Cu^++^, there appear to be differences in the coordination and bonding between these preservatives shortly after treatment. These differences can be better understood by a closer examination of each preservative system.

**Fig 4:**
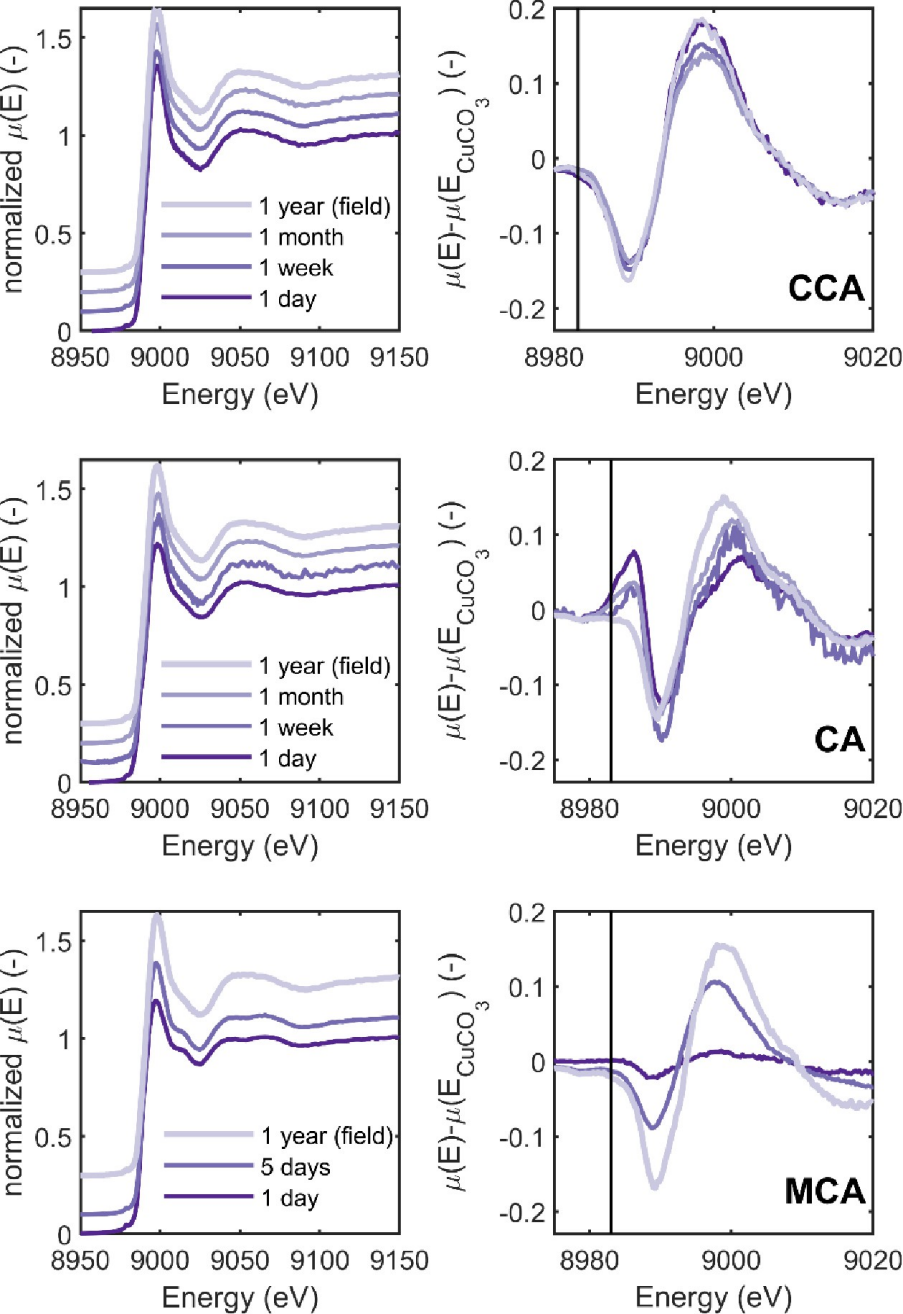
XANES patterns for three preservative systems as a function of time. The left column presents the normalized X-ray absorption coefficient; data are vertically offset for clarity. The right column presents difference spectra where the CuCO_3_ data have been subtracted from the data in the left column. From top to bottom the preservatives are chromated copper arsenate (CCA), copper azole (CA), and micronized copper azole (MCA). For MCA measurements the line shaded as “1w” was taken after 5 days and no measurement was taken at 1 month. The vertical line is at 8983 eV and used to calculate E_x_ in Eq 1.

The time course series for all preservatives is shown in Fig 4; the left column presents the normalized *μ*(*E*) spectra and the right column shows the difference spectra where the CuCO_3_ standard is subtracted from the data in the left column.

The top row of Fig 4 presents the time series of copper oxidation states for CCA. In the difference spectra, the data do not exhibit any peak at 8983 eV (highlighted with a vertical line), and in fact the curve is slightly negative at this point. This means that there is no detectable Cu^+^ in the treated wood. While some small differences between the peak at 9000 eV can be observed, there is no consistent trend with exposure time.

The XANES patterns as a function of time for CA are presented in the middle row of Fig 4. Similar to CCA, the normalized *μ*(*E*) data (left column) are nearly indistinguishable in from each other; variations between the samples can be more easily seen in the difference spectra (right column). Unlike the CCA data, the CA data shows clear differences between the data at 1 day and 1 year with time points in between those extremes showing intermediate values.

The MCA XANES patterns are given in the bottom row of Fig 4. Similar to CA, MCA exhibited changes in the XANES pattern as a function of time. However, how the spectra change as a function of time appears much different. After 1 day, the spectra are very similar to that of CuCO_3_. This can be most clearly observed in the difference spectra (right column) where the difference spectra appear to exhibit almost no deviations from zero. After 5 days, two peaks can be observed in the difference spectra, a negative peak at 8990 eV, and a positive peak near 9000 eV. Both peaks shift to higher energies and grow in amplitude in the samples that had exhibited 1 year field exposure.

**Fig 5:**
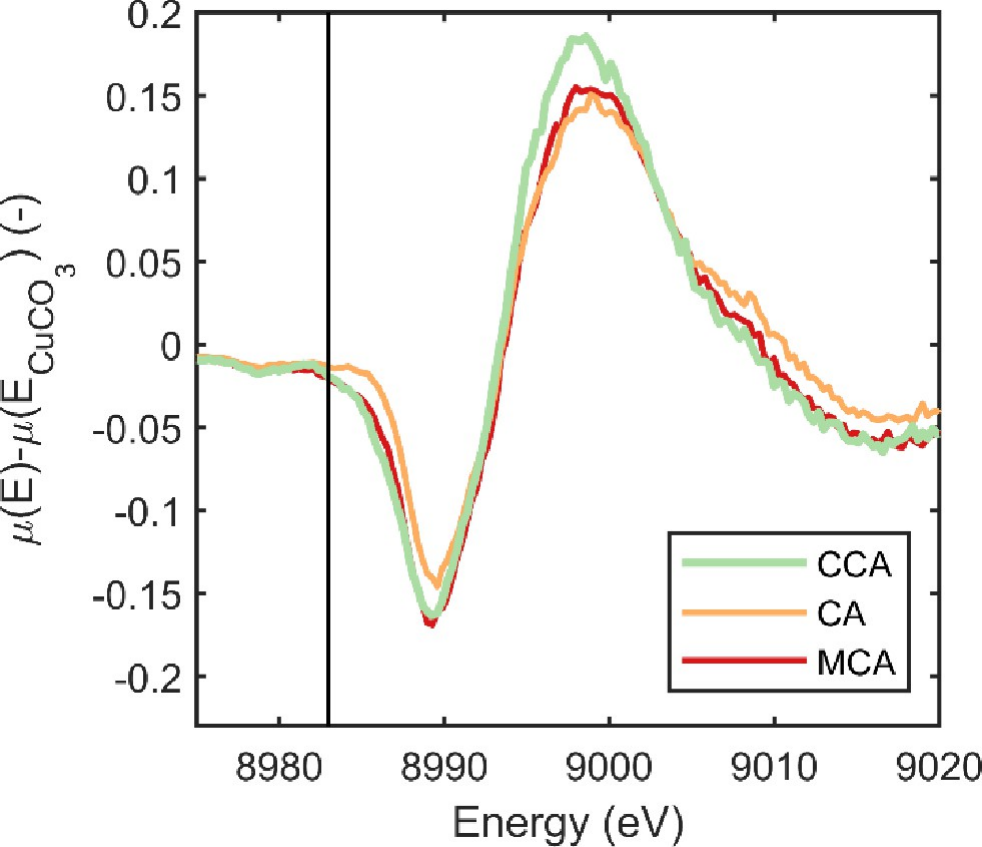
Difference spectra between the normalized *μ*(*E*) data and the CuCO3 standard after a 1 year field exposure test; the vertical line is at 8983 eV and used to calculate Ex in Eq 1.

It is interesting to note that while differences between the treatments are observable 1-day after treatment, the data at 1 year after exposure looks remarkably similar across all three treatments. This can be most easily observed in Fig 5 which plots the difference spectra after 1 year of fixation time for all treatments. None of these spectra exhibit any sign of a Cu^+^ peak at 8983 eV, and importantly the spectra all exhibit the same fingerprint. That is, when compared to CuCO_3_, the differences exhibit a negative peak at 8990 eV and a positive peak near 9000 eV. Furthermore, the magnitude of these peaks is similar across treatments. These preservative treatments start with the copper in different forms and have different fixation mechanisms, which can be seen in the first few days and weeks after treatment. However, over time, the copper within the treated wood evolves to a similar Cu^++^ species across all treatments and is all in the Cu^++^ oxidation state to the resolution limit of the technique.

It is important to discuss Fig 5 in the context of wood preservation treatments. Micronized treatments are believed to not penetrate the cell walls as well as soluble copper systems and aggregate in cell wall structures such as bordered pits [13–15, 36]. Likewise, differences have been observed between ammonia/amine based systems and acidic systems in terms of penetration and fixation especially for CCA where the rapid reaction of chromium in the wood may affect copper fixation [13]. The data in Fig 5 suggests that regardless of the treatment type, the copper eventually reaches the same binding environment with enough time in the treated wood. This is also important for the environmental impact of wood treated with micronized formulations as the studies looking at released particle size were conducted for exposures less than 5 months [9, 18]. While nanoparticle emissions were not examined in the present study, it appears that after the first year the copper in micronized copper treated wood is indistinguishable from that in soluble copper treated systems. This suggests that when impregnated into the wood environment, the acidic environment of the wood cell wall may promote solubility of the copper carbonate particles and subsequently form similar ligand interactions with the wood cell wall as more soluble copper solutions.

### Samples near corroded fasteners

The results in Fig 4 are in contrast to the results of Zelinka et al. [26, 27] where large amounts of Cu^+^ were observed in 2 µm thick sections cut near corroded fasteners. Although Zelinka et al. [26, 27] took µXANES measurements in two different regions near the corroded fastener surface, both regions showed high amounts of Cu^+^ (see Fig 1). Zelinka et al. [26, 27] interpreted this lack of difference in copper oxidation state between these regions to mean that neither region was affected by corrosion and the treatment contained Cu^+^ ions. However, the results in Fig 4 do not confirm this interpretation. Instead, the data in Fig 4 suggest that the most likely explanation for the high amounts of Cu^+^ was beam damage on the fragile, 2 µm-thick samples used at the 2-ID-D beamline.

**Fig 6:**
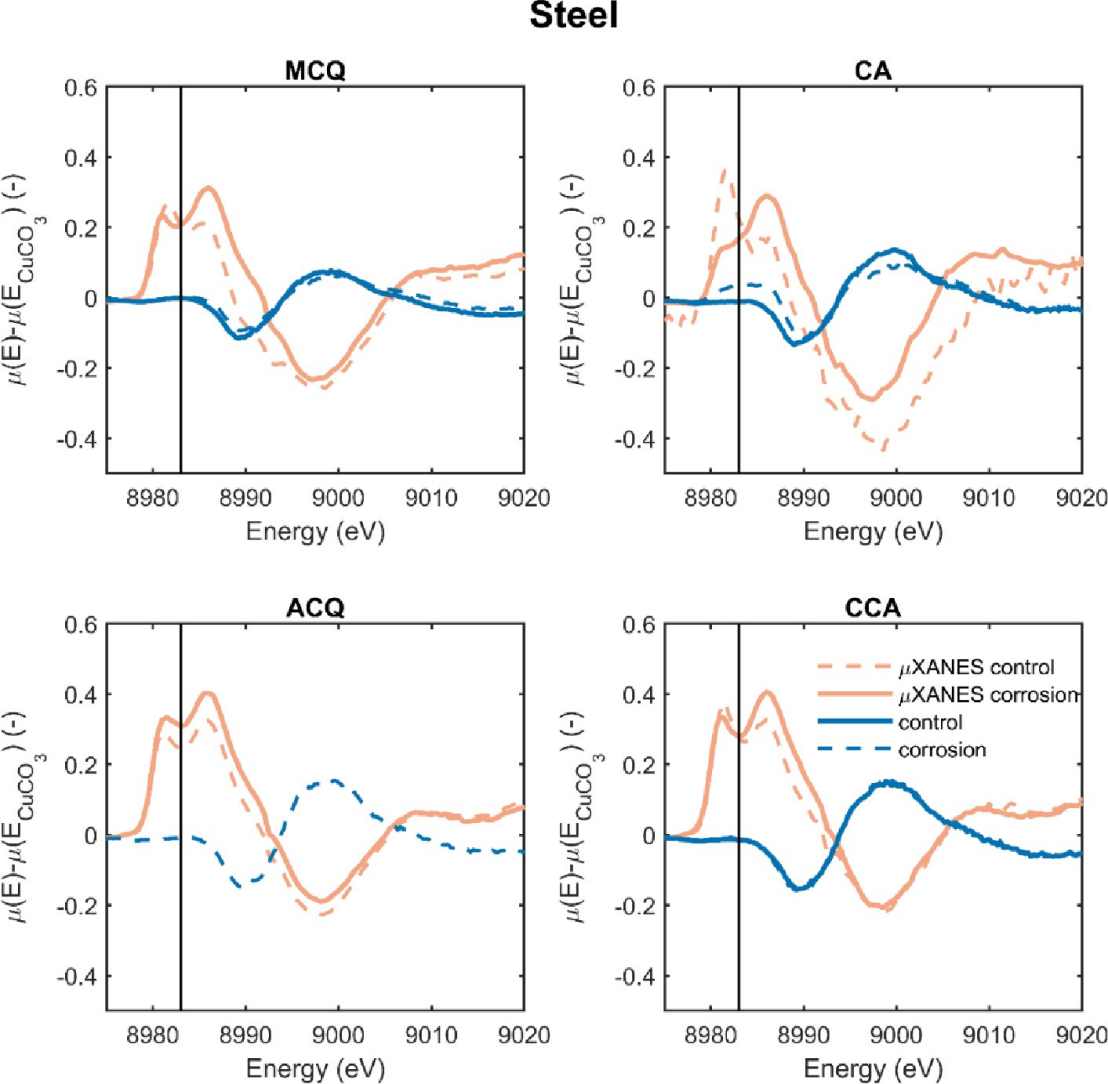
Difference spectra where the normalized X-ray absorbance of CuCO_3_ was subtracted from that collected from wood in contact with steel fasteners in a 1-year exposure test [6]. Red curves were collected as part of the current study and the blue curves (labeled “µXANES”) were collected in a previous study [26]. The vertical line is at 8983 eV and used to calculate E_x_ in Eq 1.

**Fig 7:**
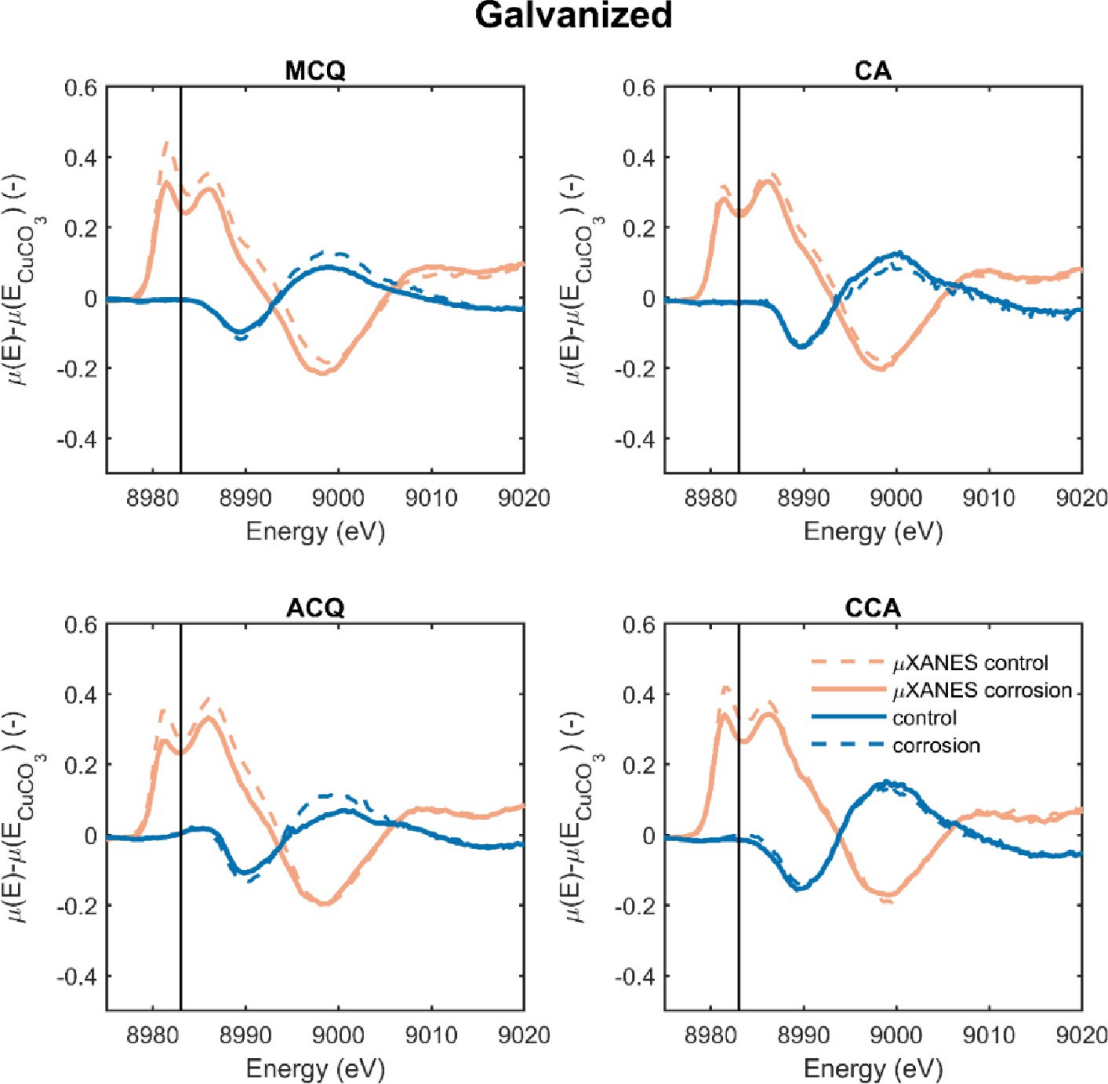
Difference spectra where the normalized X-ray absorbance of CuCO_3_ was subtracted from that collected from wood in contact with hot-dip galvanized (zinc coated) fasteners in a 1-year exposure test [6]. Red curves were collected as part of the current study and the blue curves (labeled “µXANES”) were collected in a previous study [26]. The vertical line is at 8983 eV and used to calculate E_x_ in Eq 1.

To test whether the samples of Zelinka et al. [26, 27] contained a high amount of Cu^+^, additional measurements were taken on the wood blocks used in the 1 year corrosion test [6]. These wood blocks were in contact with a carbon steel or hot dip galvanized (10–25 µm coating thickness) fasteners for 1 year at 27°C and near 100% relative humidity; further details of the exposure test are a given in the previous studies [6, 26, 27]. Measurements were taken at the surface that was in contact with the fastener and far from this surface, at the opposite edge of the block. These results are then plotted over the µXANES measurements of Zelinka et al. [26, 27] in Figs 6 and 7 for steel and galvanized steel, respectively.

Fig 6 presents difference spectra where the normalized X-ray absorbance of CuCO_3_ was subtracted from the data collected in treated wood. The µXANES measurements were taken from the previous work of Zelinka et al. [26, 27] and both the control and corrosion regions were within 200 µm of the fastener surface. From Fig 6 clear differences can be observed from the µXANES measurements and XANES measurements on the same block of wood. The control measurements taken tens of millimeters away from the corroded surface show no evidence of Cu^+^. Furthermore, with the exception of CA treated wood, the XANES measurements with the larger beam diameter cannot detect Cu^+^ even when the beam was directed to the wood surface that was in contact with the metal and showed iron staining.

Like Figs 6 and 7 presents difference spectra for the four preservative treatments; the difference is that the spectra in Fig 7 were measured on wood in contact with galvanized (zinc-coated) nails. Similar trends occur in Fig 7; the XANES measurements show no evidence of Cu^+^, and all of the µXANES measurements contain a peak near 8983 eV indicative of Cu^+^.

The data in Figs 6 and 7 show that the high amounts of the Cu^+^ in the µXANES measurements of Zelinka et al. [26, 27] were not found when examining larger pieces of wood from the same sample. This suggests that the majority of copper in treated wood is in the Cu^++^ state, even after long exposures and in the presence of corroding metals. While it is possible that Zelinka et al. [26, 27] observed Cu^+^ that had been reduced in the corrosion reaction and diffused up to 200 µm from the metal surface, a more likely explanation is that the oxidation state measured in the study was affected by beam damage caused by the focused beam on thin, 2 µm thick samples [28]. In both cases, it must be concluded that copper in treated wood is predominantly in the Cu^++^ oxidation state. Furthermore, it is important to note that regardless of the starting treatment the final Cu speciation is the same within one year.

## Conclusions

These data demonstrate that no Cu^+^ is detectable in wood treated with CCA, CA, MCA, ACQ, or MCQ at times greater than or equal to 1-day after treatment.

The bonding of copper to treated wood appears remarkably similar across all wood treatments 1-year or more after treatment. This is true for both traditional preservatives and micronized systems. In other words, there appears to be no difference after 1-year of treatment between the binding of copper to the cell wall for micronized and traditional preservative treatments.

Although large amounts of Cu^+^ were previously identified in µXANES of treated wood near corroded fasteners, this appears to be an artifact caused by beam damage or perhaps an extremely localized effect. Irrespective of the reason for the observed Cu+ in the measurements of Zelinka et al. it must be concluded that the copper in treated wood is comprised almost entirely of Cu^++^ and this does not change over long periods of time, exposure in field studies, or in the presence of corroding metals.

## Supporting information

S1 Dataset (ZIP)
