# Supplementary material for: Oxidation states of copper in preservative treated wood as studied by X-ray absorption near edge spectroscopy (XANES)
Source: PLoS One. 2022 Jan 27;17(1):e0263073. doi: 10.1371/journal.pone.0263073 (PMC8794131; doi:10.1371/journal.pone.0263073)
Supplement: S1 Dataset — (ZIP) [file pone.0263073.s001.zip › ATHENA_data_PLOSOne/Metadata.rtf]

Note- XANES data are provided in project files that can be open and analyzed with the freely available Athena [1] software. Available at http://bruceravel.github.io/demeter/documents/Athena/index.html 

1.	Ravel, B.; Newville, M. ATHENA, ARTEMIS, HEPHAESTUS: data analysis for X-ray absorption spectroscopy using IFEFFIT. Journal of synchrotron radiation 2005, 12, 537-541.
